# Modifying the Rizzoli foot model to improve the diagnosis of pes-planus: application to kinematics of feet in teenagers

**DOI:** 10.1186/s13047-014-0057-2

**Published:** 2014-12-20

**Authors:** Nicola Portinaro, Alberto Leardini, Artemisia Panou, Valerio Monzani, Paolo Caravaggi

**Affiliations:** Department of Orthopaedic and Trauma, University of Milano, Milano, Italy; Movement Analysis Laboratory, Istituto Ortopedico Rizzoli, Via di Barbiano 1/10, 40136 Bologna, Italy; Pediatric Orthopaedic Unit, Humanitas Research Hospital, Rozzano, Milano, Italy

**Keywords:** Multisegmental foot modeling, Kinematics, Children, Gait

## Abstract

**Background:**

A number of multi-segment foot protocols have been proposed to obtain measurements of clinical value. In the clinical assessment of foot pathologies and deformities, such as in the pes-planus, the frontal-plane alignment of the calcaneus and the dynamic properties of the medial longitudinal arch are critical parameters though often neglected by the majority of foot protocols. The aim of the present work is to modify an established foot protocol to obtain static and kinematic measures more consistent with corresponding clinical observations. Moreover, while many papers have reported kinematic data from varying populations, few investigations have focussed on young participants from same-age cohorts.

**Methods:**

A 6-camera motion capture system was employed to track the shank, rear-, mid- and fore-foot segments in the left and right leg of 10 children (13.1 ± 0.8 years) during gait. Three markers were attached to each segment thus allowing for triplanar motion of five joints to be described according to the Rizzoli Foot Model. An additional marker was attached to the posterior bottom of the calcaneus to enhance measurement of frontal-plane orientation. Description of the medial longitudinal arch angle was redefined to be more consistent with rearfoot orientation and to common clinical assessments. A novel 3-marker description of the hallux segment was implemented to improve robustness in calculating 1^st^ metatarso-phalangeal joint rotations.

**Results:**

Foot segments kinematics showed good inter- participant repeatability and overall consistency with previous similar reports. 15 out of 20 feet showed neutral or slightly valgus orientation of the calcaneus. Relatively large medial longitudinal arch angles (mean 186 ± 16 deg) were found in the present young population. Both measurements were reasonably in accordance with the relevant clinical observations of these feet.

**Conclusions:**

Modifications to a widely used multisegmental foot kinematic model were implemented to improve robustness and consistency with relevant clinical observations. A detailed description of foot joints motion during barefoot walking in a population of 13-year old children with apparent flat feet has been presented, which may provide useful information to investigate the development of gait in children and the diagnosis of flexible flat foot.

## Background

Increased interest in multi-segment foot kinematics in-vivo using stereophotogrammetry is documented in the recent literature [1,2]. The description, limitation and applicability of a large number of different models have been reported. Several studies have investigated foot joint kinematics in healthy participants and in patient cohorts of varying ages, while fewer have addressed multi-segment foot kinematics in children [3-9].

A multi-segment foot model proposed recently (Rizzoli Foot Model, RFM) [10] has been validated by various research teams in different populations [11-14], utilized in biomechanical and clinical studies [2,15,16], and proved to be the most reliable when directly compared to others [17,18]. In accordance with standard recommendations [19,20], the model allows for a detailed description of foot kinematics throughout the gait cycle by measuring three-dimensional (3D) motion of the talocrural, Chopart and Lisfranc joints, as well as eight planar rotations relevant to the clinical setting. The number and location of the markers were chosen to be tracked with camera configurations typical for full-body gait analysis while maintaining minimum encumbrance to the participant. However, the initial utilization of this model has revealed the need for more robust calculations, such as those relative to the 1^st^ metatarso-phalangeal joint (1MPJ) rotations, and for measurements more consistent with clinical observations, particularly those associated to common foot deformities as in the pes-planus. As for the latter, the most critical variables in the diagnosis are the frontal-plane orientation of the calcaneus and the angle representing the medial longitudinal arch (MLA). In fact, because of the position of the anatomical landmarks used to track the calcaneus in [10], the measured frontal-plane angle in up-right neutral position in normal feet was found not to be consistent with the clinical observations of 0 to 5 degrees in valgus [21-23]. Additionally, the original two marker-based vectors used to estimate the MLA angle did not appear to resemble exactly the corresponding traditional X-ray based measures, commonly known in the clinical setting as the Moreau and Costa-Bertani angle. Modifications to the original kinematic protocol had to be sought to better evaluate the most common deformities typical of the pes-planus.

The aim of the present work was to apply a modified RFM to the kinematic analysis of feet in a special population of young children, which presented deformities common to flat foot but that did not result in an indication for surgical intervention. Such population provided an ideal scenario to assess measurements of not-fully developed foot arches with respect to the modifications. The implications of the protocol in relation to the new dataset have also been discussed.

## Methods

Ten young participants (5 males 5 females; shoe size range 37 – 44; age 13.1 ± 0.8 years; height 162.2 ± 10.6 cm; body mass 48.4 ± 10.7 kg), who scheduled an appointment at the authors’ clinic for a medical examination regarding possible pes-planus diagnosis, were enrolled in the study. According to the current clinical protocol, which does not regard the shape of the MLA as indication of pes-planus deformity, all participants had normal feet presenting a hindfoot frontal-plane inclination lower than 7 deg in valgus. Parents’ informed consent was given for the kinematic data collection. Ethical approval for the study was provided by the ethical board of the authors’ Institute Comitato Etico Indipendente of Istituto Clinico Humanitas.

**Figure 1:**
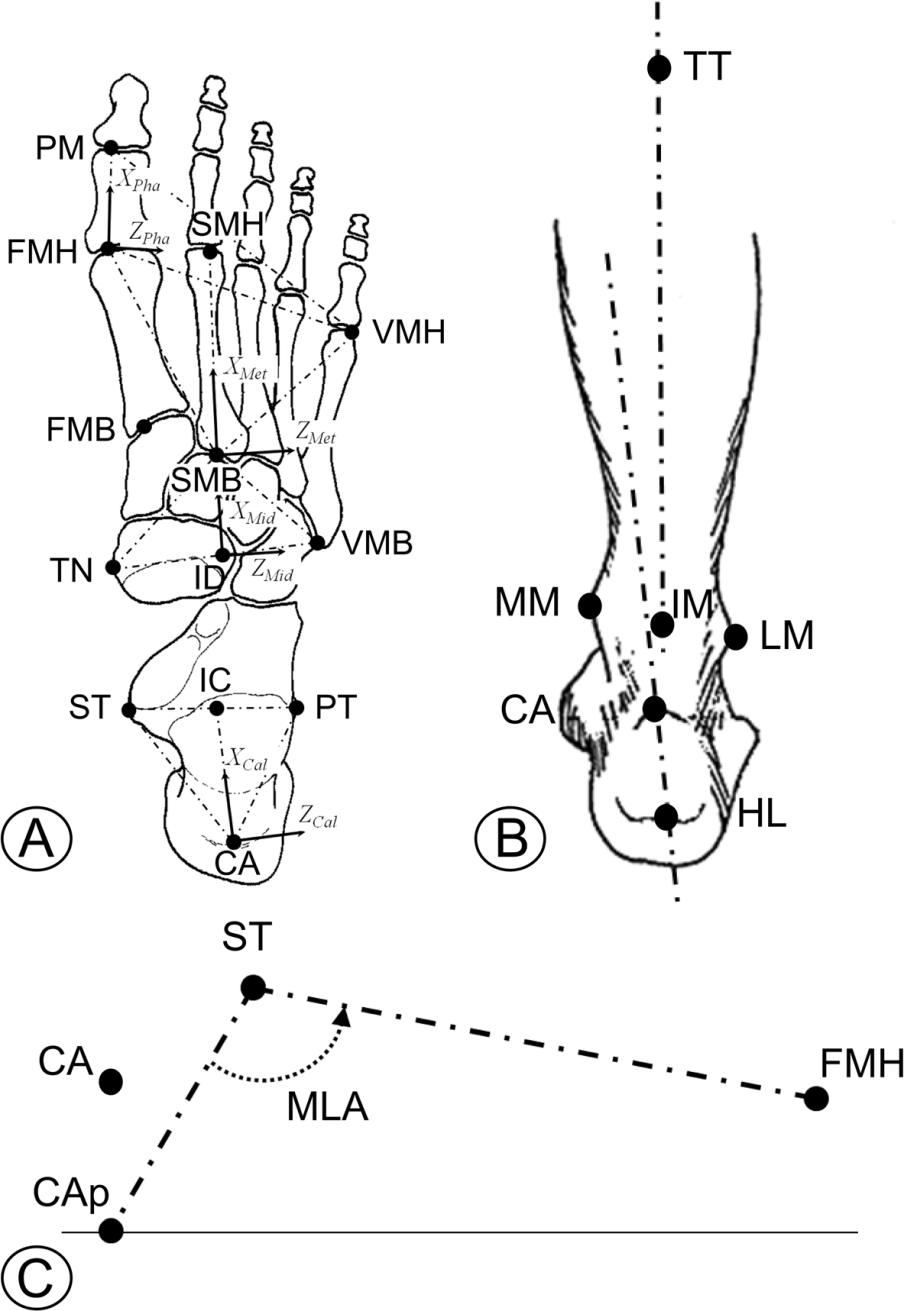
**Diagram of the three modifications to the foot protocol. A)** Definitions of the four rigid segments according to relevant anatomical landmarks (full list in Leardini et al. [10]). X and Z axes (solid arrows) of the anatomical reference frames are shown together with corresponding transverse planes (dash-dot triangles), including the new one for the Phalanx. **B)** Definition of the improved frontal-plane angle of the calcaneus with respect to the shank, according to the position of the additional marker HL. **C)** Scheme for the calculation of the planar MLA angle in the sagittal plane of the foot, where CAp is the projection of the CA marker on the ground.

The first modification to the original RFM is related to the definition of a new anatomical reference frame for the proximal phalanx of the hallux (Figure 1A). This was established by the location of three markers: PM on the head of the proximal phalanx; FMH, on the head of the first metatarsal, and VMH, on the head of the fifth metatarsal. The vertical axis (Y axis) was defined as the vector orthogonal to the plane passing through the three markers, the antero-posterior axis (X axis) was the line segment FMH-PM and the medio-lateral axis (Z axis) was the vector product of the other two. A joint coordinate system [24] was defined to determine 3D angles between the hallux and the metatarsus segments (Met-Hal joint), the latter being identified by the position of FMH, VMH and SMB, as in the original model [10].

The second modification concerned the Eve/Inv offset angle of the calcaneus with respect to the shank (Figure 1B). This modification, which concerns the static calibration, entailed the use of an additional marker (HL) on the most distal point of the attachment area of the Achilles tendon on the calcaneus. A new frontal-plane offset for the Sha-Cal joint was defined as the angle between the line segment CA-HL projected in the frontal plane of the shank, and the shank vertical axis in up-right double-leg static posture.

A third modification involved the MLA angle (Figure 1C). This was here defined as the angle between the projections, into the sagittal plane of the foot, of the line segments between ST and FMH and between ST and CAp. The latter point is identified by the vertical projection of CA on the ground in up-right posture, and it is then tracked during walking by a technical reference frame based on the three markers on the calcaneus. Such modification was intended to achieve a better consistency with the rearfoot orientation and with clinical and radiographical definitions. By doing so, the newly defined MLA angle may be described as a compromise between the clinical Moreau and Costa-Bertani angle [25] and the calcaneal pitch.

With exception of one minor modification described above, the marker-set utilized was that described in Leardini et al. [10] (Figure 1). Motion data collection implied a few seconds in up-right double-leg posture, and three trials of barefoot walking at self-selected speed. Marker trajectories were collected by a six-camera motion capture system (Bonita B10, Vicon Motion Systems Ltd, Oxford, UK) at 100 Hz. Motion of the markers on the calcaneus and on the forefoot were used to identify the gait cycle [26]. 3D joint rotations, according to the standard Grood and Suntay convention [24], were calculated between the shank, i.e. tibia and fibula, and the calcaneus (Sha-Cal), between the calcaneus and the mid-foot (Cal-Mid), and between the mid-foot and the metatarsus (Mid-Met). The rotations of the entire foot with respect to the shank (Sha-Foo), and of the metatarsus with respect to the calcaneus were also calculated (Cal-Met). Dorsi-/plantar-flexion (Do/Pl), abduction/adduction (Abd/Add) and eversion/inversion (Eve/Inv) rotations were calculated at each joint, in the sagittal, frontal and transverse plane respectively. Sagittal-plane angles of the 1^st^, 2^nd^, and 5^th^ metatarsal bones in the laboratory frame, and transverse-plane angles of the 1^st^ and 5^th^ metatarsals with respect to the 2^nd^ were also calculated.

## Results

**Figure 2:**
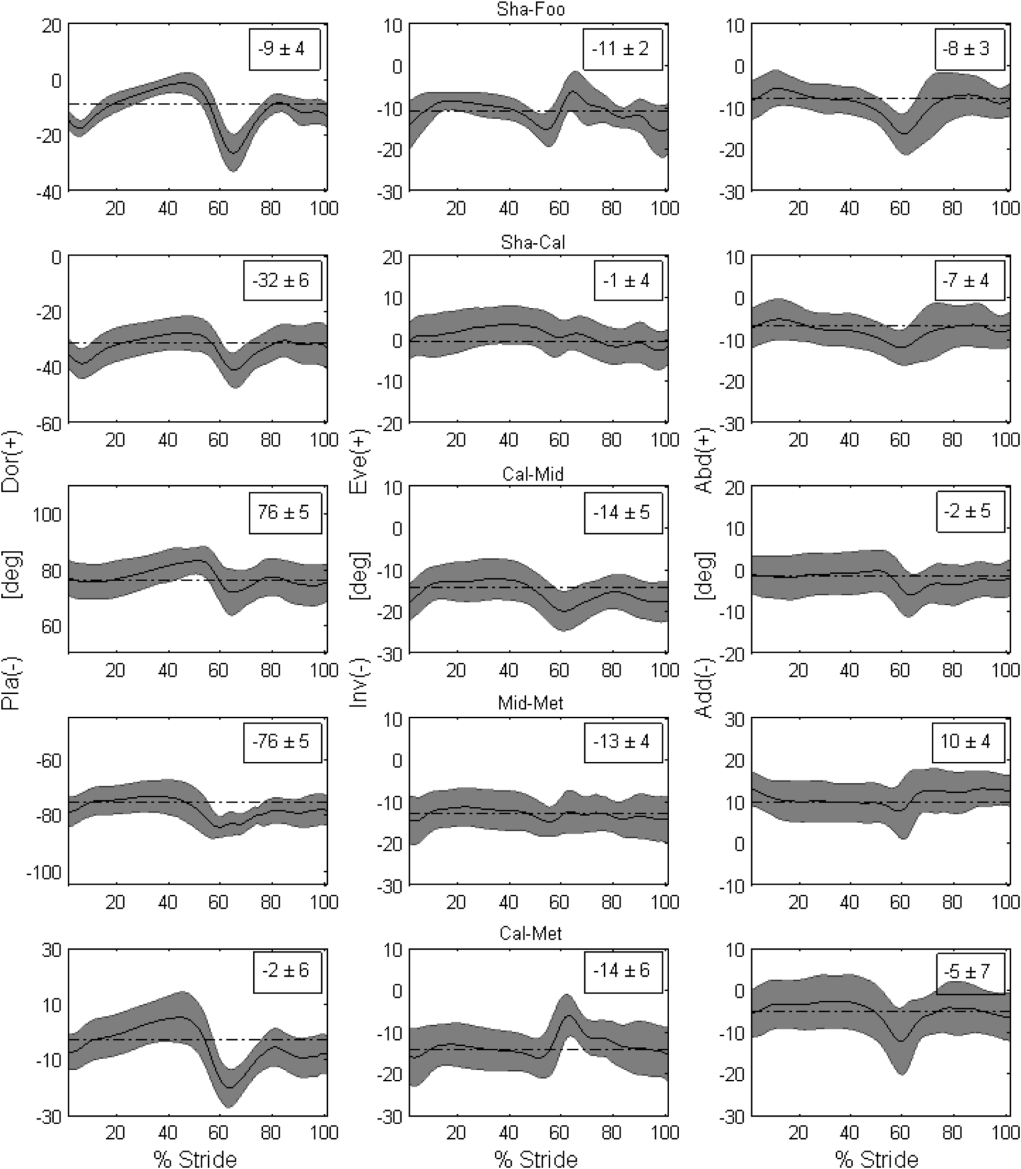
**Temporal patterns of 3D joint rotations.** Left to right, the patterns of rotation in the sagittal, frontal and transverse planes for each of the five joints analyzed (Sha-Foo, Sha-Cal, Cal-Mid, Mid-Met, Cal-Met) in the right foot and leg. Mean (solid line), plus and minus a standard deviation (grey band), were calculated across 30 walking trials, 3 repetitions for each of the 10 participants. The corresponding mean value obtained in the static up-right posture is superimposed to the temporal profile (dash-dot line segment) and reported numerically within each plot.

**Figure 3:**
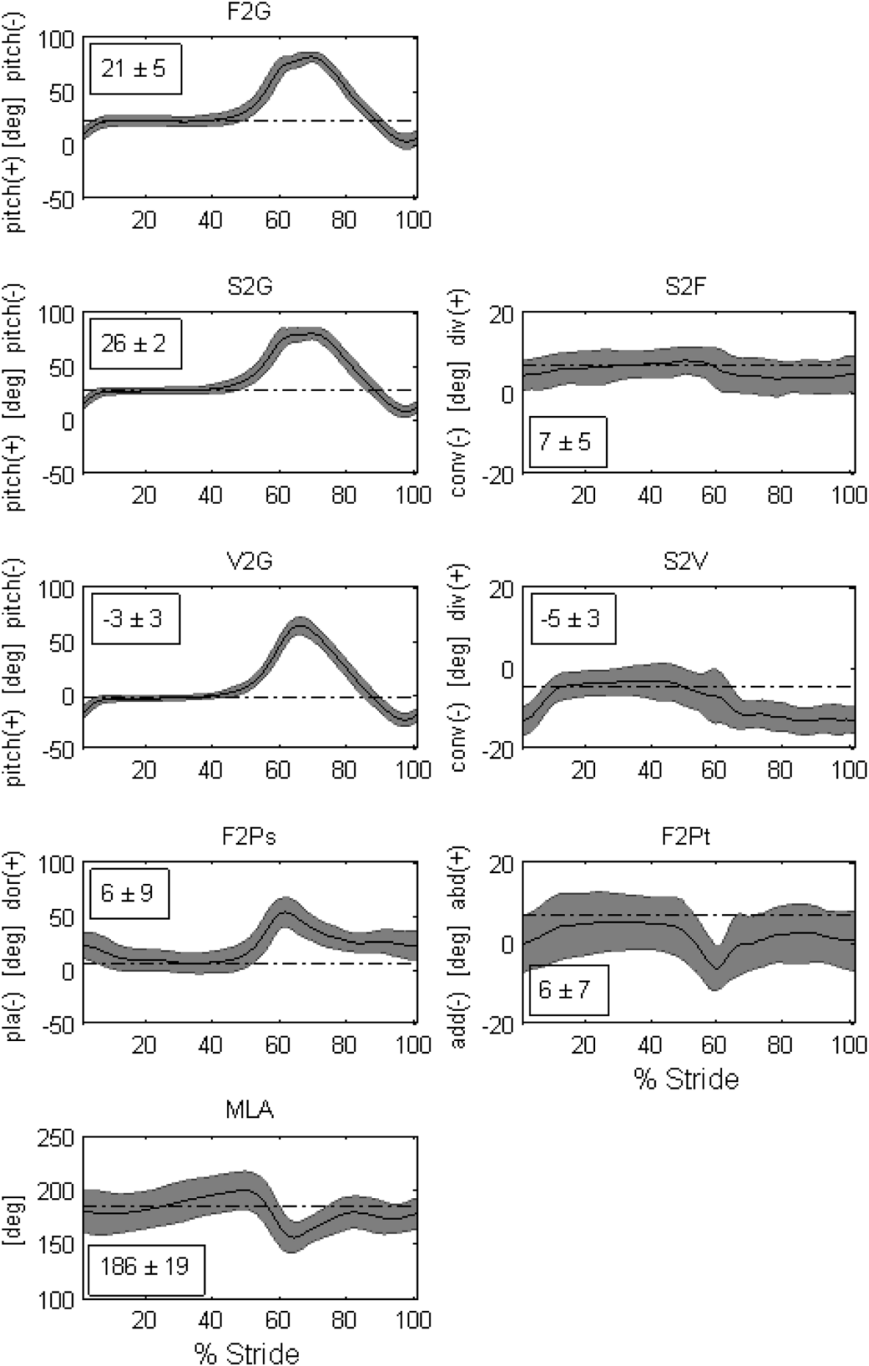
**Temporal patterns of planar angles.** Where, from top to bottom: F2G is the sagittal-plane angle of the 1^st^ metatarsal bone to the ground; S2G is the sagittal-plane angle of the 2^nd^ metatarsal bone to the ground; S2F is the transverse-plane angle between 1^st^ and 2^nd^ metatarsal bones; V2G is the sagittal-plane angle of the 5^th^ metatarsal bone to the ground; S2V is the transverse-plane angle between 5^th^ and 2^nd^ metatarsal bones; F2Ps is the sagittal-plane angle between 1^st^ metatarsal bone and proximal phalanx; F2Pt is the transverse-plane angle between 1^st^ metatarsal bone and proximal phalanx, and MLA is the angle between CAp-ST and ST-FMH projected into the sagittal plane of the foot. Mean (solid line), plus and minus a standard deviation (grey band), were calculated across 30 walking trials, 3 repetitions for each of the 10 participants. The corresponding mean value obtained in the static up-right posture is superimposed to the temporal profile (dash-dot line segment) and reported numerically within each plot.

Consistent patterns of 3D joint rotations (Figure 2) and of planar angles (Figure 3) were observed across all participants for both legs. Mean standard deviation over the gait cycle ranged between 3.9 and 7.2 deg for all the 3D joint rotations and between 3.9 and 16.5 deg across all planar angles. Kinematics were also in good agreement with corresponding data obtained with very similar anatomical definitions [9,15].

The novel 3D description of the 1MPJ resulted in robust calculation of rotations (F2Ps and F2Pt, Figure 3), where no numerical singularities have arisen when processing the recorded gait cycles. In the double-leg stance static position, across the 20 feet analyzed (10 left, 10 right), 7 feet showed neutral orientation in the frontal plane (between −2 and 2 deg), 5 showed varus orientation (inversion larger than 2 deg), and 8 showed valgus orientation (eversion larger than 2 deg). When compared to the original calculation [10] of the frontal-plane offset angle between shank and calcaneus, 9 feet showed a more valgus orientation (range 2.3 – 14.7 deg) and 9 feet showed a more varus orientation (range 2.1 – 9.0 deg). In accordance with the clinical observation, the inter-participant mean MLA angle in double-leg stance was 183 ± 16 deg (range 153–206 deg) across the 20 feet. No major difference in MLA kinematic pattern (Figure 3) was observed with respect to what measured with the previous definition [10] in a healthy older population.

## Discussion

The continuous need for addressing special clinical problems of the foot by kinematics measurements, is pushing research towards the design of new or enhanced multi-segment models, as well as extending the availability of reference data for different pathologies and age groups. An established and validated model [10] was here modified to increase its robustness but mainly to provide measures more consistent with clinical definitions and observations, particularly in the field of functional assessment of the pes-planus.

A new anatomical reference frame was established for the hallux, which is now modeled as a three-dimensional segment as to improve the robustness of the calculation relative to 1MPJ rotations. This modification to the original model was made necessary also to overcome mathematical singularities arising in the trigonometric calculation for large rotation angles at this joint, such as those arising in the push-off phase. However, this modification does not permit for axial rotation to be correctly calculated for this joint, rather Do/Pl and Abd/Add can now be estimated in a more reliable way.

MLA angle was calculated in a more clinically meaningful way, with only a minor difference to the original protocol. In relation to its novel definition, which entails the use of the projection of the calcaneus’ posterior marker on the ground, no evident differences in the mean temporal profile was detected in comparison to that recorded using the previous definition [10]. The measured MLA angle in static up-right posture was larger than 180 deg in about 50% of the feet. This was consistent with the clinical observation of reduced arch height across the present population of young participants, for which not-fully-developed and/or low-arched feet are considered to be physiological. In fact, the reduced arch height observed from simple visual inspection, typical in pes-planus, was the main reason for these feet to require further clinical assessment.

The frontal-plane offset angle of the calcaneus with respect to the shank, i.e. the calcaneal varus or valgus orientation, was altered to provide a more anatomical representation of this important clinical parameter. A recent review on current marker-sets for multisegment analysis of foot motion [27] has highlighted how the resultant coordinate system of the hindfoot is tipped slightly in inversion in the RFM. The inconsistency between this calculation and the clinical observation is due to the common anatomical higher position of ST in relation to PT. In other words, a more varus orientation of the hindfoot with respect to the tibia, associated to the definition of the relevant anatomical frames, is in contrast with the typical clinical observation of valgus or neutral calcaneus based on the clinical appearance of the posterior aspect of the calcaneus [21-23]. This was explicitly estimated in each participant by adding an additional marker at the base of the calcaneus (HL, Figure 1B) during the static calibration. Preliminary experiments performed by the same authors on a synthetic model of the lower limb have shown that a measurement much closer to the physiological alignment of the calcaneus can indeed be achieved by using the new definition of calcaneal offset. The suggested correction to the RFM resulted in neutral or valgus orientation of the calcaneus for 15 out of 20 feet analyzed, thus 25% of the measurements did not match the clinical valgus or neutral orientation. Such discrepancy may be accounted for by the tibial axis malicious orientation observed in some of the participants. In fact, by measuring the frontal-plane calcaneus orientation with respect the tibial axis, a varus orientation of the calcaneus may be measured in those patients presenting genu valgum, i.e. femur in neutral position and tibia adducted and internally rotated. While calculation of the newly defined offset on a larger and older population cohort may be performed to fully confirm the better matching with the anatomical alignment as observed in-vitro, the authors believe that a more clear definition for the position of the marker at the base of the calcaneus should be sought to achieve greater consistency with the in-vitro measurements and the clinical observation.

## Conclusions

The present results are in good agreement with established biomechanical knowledge and common clinical observations. In general, the kinematic patterns reported here are consistent with those reported in other studies that used the marker-set and the definitions from Leardini et al. [10], i.e. [2,11-16,18,28]. With the exception of one additional marker on the calcaneus, these adjustments enhanced the original protocol with no significant alterations to the marker-set, to the biomechanical model, and to the overall time required by the data collection and analysis. In conclusion, the present study reports improvements of a previous protocol for multi-segment foot kinematics, and provides a useful reference kinematic dataset for the whole gait cycle, which may be employed in clinically-oriented analyses of younger participants.
